# Production of oligosaccharides and biofuels from *Miscanthus* using combinatorial steam explosion and ionic liquid pretreatment

**DOI:** 10.1016/j.biortech.2020.124625

**Published:** 2021-03

**Authors:** Rakesh Bhatia, Jai B. Lad, Maurice Bosch, David N. Bryant, David Leak, Jason P. Hallett, Telma T. Franco, Joe A. Gallagher

**Affiliations:** a Institute of Biological, Environmental and Rural Sciences (IBERS), Aberystwyth University, Plas Gogerddan, Aberystwyth SY23 3EE, UK; b ARCITEKBio Ltd, Aberystwyth Innovation and Enterprise Campus (AIEC), Aberystwyth University, Plas Gogerddan, Aberystwyth SY23 3EE, UK; c Department of Biology and Biochemistry, University of Bath, Claverton Down, Bath BA2 7AY, UK; d Department of Chemical Engineering, Imperial College London, Exhibition Road, London SW7 2AZ, UK; e Faculty of Chemical Engineering, University of Campinas (UNICAMP), Campinas, São Paulo 13083-852, Brazil

**Keywords:** *Miscanthus*, Steam explosion, Ionic liquid, Oligosaccharides, Biorefinery

## Abstract

Pretreatment strategies are fundamental to effectively deconstruct lignocellulosic biomass and economically produce biofuels, biomaterials and bio-based chemicals. This study evaluated individual and combinatorial steam explosion (SE) and ionic liquid (IL) pretreatments for production of high-value oligosaccharides from a novel seed-based *Miscanthus* hybrid (*Mx2779*). The two ILs used for pretreatment were triethylammonium hydrogen sulphate [TEA][HSO_4_] and 1-ethyl-3-methylimidazolium acetate [C_2_mim][OAc]. The results showed that each pretreatment leads to distinct effects on the fragmentation (cellulose and xylan dissolution, delignification, deacetylation) and physicochemical modification (cellulose and lignin properties) of lignocellulose. This, in turn, dictated enzymatic hydrolysis efficiencies of the cellulose pulp to glucose or gluco-oligosaccharides for downstream applications. Our findings suggest that the stand-alone SE or [C_2_mim][OAc] pretreatments may offer cost advantages over [TEA][HSO_4_] through the production of oligosaccharides such as xylo- and gluco-oligosaccharides. This study also highlights technical and economic pretreatment process challenges related to the production of oligosaccharides from *Miscanthus Mx2779* biomass.

## Highlights

• For oligosaccharides production, SE and [C_2_mim][OAc] pretreatment is advised.
• 90% (w/w) of initial biomass xylan dissolved to XOS via [C_2_mim][OAc] pretreatment.
• Up to 50% glucan to GOS conversion using commercial *endo*-1,4-β-D-glucanases.
• 33% extra profit for SE + [TEA][HSO_4_] compared to [TEA][HSO_4_] pretreatment.
• XOS could contribute to economic viability of an integrated biorefining process.

## 1 Introduction

The perennial C_4_ grass *Miscanthus* is a dedicated biomass crop that grows over a broad range of environmental and climatic conditions, with high yield potential on marginal and erosive land resulting from its high-water use efficiency and nutrient sequestering ability. Recent breeding programmes at Aberystwyth University in the UK have resulted in the development of novel high-yielding seed-based *Miscanthus* hybrids to ensure feasible commercial upscaling of *Miscanthus* across Europe. These hybrids are being investigated as an agronomically and economically viable lignocellulosic biomass resource for industrial production of renewable and sustainable biofuels and commodity bio-based products (Clifton-Brown et al., 2019).

*Miscanthus* is typically harvested following senescence, and the lignocellulose component of the material consists primarily of cellulose (~40 to 60 %), hemicellulose (~20 to 40 %) and lignin (~10 to 30 %) (Brosse et al., 2012). The two main polysaccharide fractions, cellulose and hemicellulose, can undergo chemical and/or microbial conversion into biofuels, chemicals, food additives or materials. Moreover, to expand the cost competitiveness of a lignocellulose-based biorefinery and for complete biomass utilisation, lignin could either provide heat and/or power generation or a stream for additional high-value products including chemicals, adhesives, carbon fibres and bioplastics (McCann and Carpita, 2015, Ragauskas et al., 2014). One of the main challenges impeding efficient biomass conversion lies within the intrinsic structural properties and chemical complexity of lignocellulose that forms the basis of biomass recalcitrance to its deconstruction. Important recalcitrance factors associated with biomass conversion include the crystalline structure and degree of polymerisation (DP) of cellulose as well as hemicellulose and lignin content and composition.

An essential processing step to overcome lignocellulose recalcitrance for effective biomass conversion has been to deploy structural disruption via physical (milling, grinding), chemical (acid, alkali and ionic liquid), biological (biomass-degrading microorganisms) or physicochemical (liquid hot water, steam explosion and wet oxidation) pretreatments. These pretreatments have different effects on the fragmentation and physicochemical modification of lignocellulose, which in turn dictate the environmental and economic feasibility of commercial production of bio-based products (Galbe and Wallberg, 2019). Over the past decade, ionic liquids (ILs) have proven their utility as pretreatment solvents for the fractionation and dissolution of lignocellulose (Halder et al., 2019, Usmani et al., 2020). The effects of IL pretreatment vary from hemicellulose and lignin extraction through to decrystallisation and/or reduction in the DP of cellulose, thereby lowering native biomass recalcitrance. Such effects are dependent on the nature of the ILs (protic or aprotic), process conditions (temperature, time, biomass loading and particle size) and specific interactions of ILs with lignocellulosic biomass (i.e., grasses, hard and softwoods). However, the high operational costs (even in bulk), need for energy-efficient and cost-effective methods for recovery and reusability of ILs, the viscosity of ILs as well as equipment handling, are amongst the concerns for their industrial application. Steam explosion (SE) is a cost-effective (low recycling costs, low energy requirement and limited use of corrosive chemicals), commercially scalable and environmental friendly pretreatment that can pre-process lignocellulose to reduce biomass particle size and likewise result in the dissolution of hemicellulose and disruption of the native cellulose crystalline structure (Chen and Liu, 2015).

Despite reports on SE or ILs pretreatment of *Miscanthus*, there have been few studies investigating the benefits of applying an integrated SE and IL pretreatment process strategy for the bioconversion of cellulose, hemicellulose and lignin streams into added-value chemicals and products (Liu et al., 2016, Peng et al., 2014). In particular, the production of oligosaccharides, such as gluco-oligosaccharides (GOS) and xylo-oligosaccharides (XOS) from abundant and renewable lignocellulosic residues, is gaining commercial interest due to their increasing use as ingredients in the food and beverage, animal feed, pharmaceutical and materials industries (Santibáñez et al., 2021). Therefore, efficient oligosaccharides production technologies require an assessment before they can effectively be implemented in biorefinery processes. To fill this gap, the present study investigated combinatorial steam explosion (SE) and ionic liquid (IL) pretreatments of a novel seed-based *Miscanthus* hybrid (*Mx2779*), and its effects on biomass conversion metrics including hemicellulose hydrolysis into XOS, delignification, deacetylation, cellulose recovery and properties for controlled enzymatic hydrolysis into glucose and gluco-oligosaccharides (GOS). Herein, two widely used and distinct ILs were chosen, triethylammonium hydrogen sulphate [TEA][HSO_4_] (selectively dissolves lignin and hemicellulose) and 1-ethyl-3-methylimidazolium acetate [C_2_mim][OAc] (swells cellulose and dissolves lignin). The efficacy of ILs before and after SE pretreatment were considered for lignocellulosic deconstruction and preliminary techno-economic evaluation to identify cost-effective production of oligosaccharides and various other bio-based products from *Miscanthus Mx2779*.

## 2 Materials and methods

### 2.1 Materials

*Miscanthus Mx2779*, also known as GNT-14, is a novel rapidly multiplied seeded interspecies hybrid (*Miscanthus sinensis × M. sacchariflorus*) bred in Aberystwyth in 2013. *Mx2779* material was used and prepared for SE pretreatment as previously described (Bhatia et al., 2020). A representative portion of the untreated *Mx2779* material was prepared for IL pretreatment per technical report NREL/TP-510-42620 (Hames et al., 2008). Biomass moisture content was determined per technical report NREL/TP-510-42621 (Sluiter et al., 2008a). Triethylammonium hydrogen sulphate [TEA][HSO_4_] was synthesised as described by Gschwend et al., 2016. 1-ethyl-3-methylimidazolium acetate ([Emim][Ac]), also known as [C_2_mim][OAc], was purchased (Sigma-Aldrich).

### 2.2 Biomass pretreatment

SE pretreatment was carried out as previously described (Bhatia et al., 2020). Following SE, pretreated solids were washed with de-ionised H_2_O, dried and processed per technical report NREL/TP-510-42627 (Sluiter et al., 2008c). For sequential SE + IL pretreatment, the SE pretreated solids were taken from our previous pilot-scale study (Bhatia et al., 2020). For [TEA][HSO_4_] pretreatment, 10 g of IL [TEA][HSO_4_] containing 20 % (w/w) water and 1 g of untreated or SE pretreated biomass (biomass to solvent ratio of 1:10 g/g) was used according to a standard protocol (Gschwend et al., 2016). Briefly, [TEA][HSO_4_] pretreatment was carried out in triplicates on biomass solids at 120 °C for 8 h. After pretreatment, the pulp-IL slurry was subjected to ethanol washes followed by an ethanol Soxhlet extraction step. The cellulose-rich pulp was recovered by air-drying, weighed, and moisture content determined per technical report NREL/TP-510-42621 (Sluiter et al., 2008a). Lignin was precipitated from [TEA][HSO_4_] with de-ionised H_2_O and recovered by overnight drying in a vacuum oven at 45 °C for lignin mass balances.

For [C_2_mim][OAc] pretreatment, 300 mg of untreated or SE pretreated biomass (3 % w/w biomass loading) was mixed with 9.7 g of [C_2_mim][OAc] and pretreated at 160 °C for 3 h without stirring in 90 mL pressure tubes (Ace Glass) (Cheng et al., 2011). After pretreatment, samples were transferred to 50 mL Falcon tubes, 35 mL of hot de-ionised H_2_O was added and tubes were vigorously vortexed. The pulp was recovered by centrifugation at 3,500 rpm for 10 min. The supernatant was removed, collected, and recovered pulp residue was washed repeatedly with 35 mL of hot de-ionised H_2_O (at least four times), centrifuged and then freeze-dried for 48 h before analysis. [C_2_mim][OAc] pretreatment was performed in triplicates. An aliquot of the collected wash supernatant was subjected to end-hydrolysis with 4 % H_2_SO_4_ for sugar content and composition (monosaccharides and oligosaccharides) per technical report NREL/TP-510–42623 (Sluiter et al., 2008b).

### 2.3 Structural carbohydrates and Klason lignin

Compositional analysis of untreated and pretreated solids was determined per technical report NREL/TP-510-42618 (Sluiter et al., 2012) and monosaccharides were quantified as described previously (Bhatia et al., 2020). The percentage of structural carbohydrates and Klason lignin (acid-insoluble and acid-soluble lignin) was calculated on a dry matter (DM) basis. Extractives and ash were also determined and reported as others. Data are reported as means ± standard deviation (n ≥ 3).

### 2.4 Fourier-transform Infrared Spectroscopy (FTIR)

FTIR analysis of biomass was performed using a Thermo Nicolet iS50 FTIR spectrometer. Using 2 mg of milled sample (<80 µm), FTIR spectra were recorded in duplicates and in the range 4000–400 cm^−1^ with a resolution of 4 cm^−1^ and eight scans per sample and corrected for a baseline before data analysis.

### 2.5 Enzymatic saccharification

Enzymatic hydrolysis of untreated and pretreated solid residues was based on the technical report NREL/TP-5100-63351 (Resch et al., 2015) using Cellic CTec2 (30 % w/w g enzyme/g glucan) (Novozymes) or *endo*-1,4-β-D-glucanase (*Bacillus amyloliquefaciens*) (1.5 or 18 U/mL) (Megazyme). Briefly, biomass equivalent to 14 mg (1 % w/v biomass loading) was transferred to a 2 mL microcentrifuge tube and 42 µL of 1 M sodium citrate buffer (pH 5) with 5.6 µL of 5 % sodium azide was added for Cellic CTec2 assays. In contrast, 42 µL of 1 M sodium phosphate buffer (pH 6) containing 1 mg/mL BSA with 5.6 µL of 5 % sodium azide was used for *endo*-1,4-β-D-glucanase assays. The total volume in each tube was brought to 1.4 mL with de-ionised H_2_O before the enzyme was added. The tubes were incubated horizontally for 24 to 72 h in a shaker set at 50 °C (200 rpm) and the hydrolysis reaction was stopped by boiling samples at 100 °C for 10 min. Samples were analysed for monosaccharides or oligosaccharides yield by HPAEC (Bhatia et al., 2020). The percent glucan conversion to glucose or gluco-oligosaccharides (GOS) was reported on a DM basis as means ± standard deviation (n ≥ 3) and calculated as described elsewhere (Resch et al., 2015). The hydrolysis factor (*H*) used were 0.90 for glucose, 0.95 for cellobiose, 0.96 for cellotriose and 0.97 for cellotetraose.

### 2.6 Techno-economic estimates of SE and IL pretreatment processes

The IL pretreatment process considerations were based on previous studies (Baral and Shah, 2016, Brandt-Talbot et al., 2017). The SE pretreatment process was divided into seven primary sections, namely biomass preparation, SE pretreatment, enzymatic hydrolysis with *endo*-xylanases, XOS recovery, simultaneous saccharification and fermentation (SSF), ethanol recovery and combined heat and power (CHP) (Baral and Shah, 2017, Lopes et al., 2019). Material costs such as the price of feedstock and other primary input materials including electricity, water, IL solvents, anti-solvents and enzymes were gathered from existing literature and are summarised in Table 1. Since the scope of the study was to assess the production of oligosaccharides and other value-added bio-based products from different pretreatments, detailed capital investment and operational costs associated with XOS and GOS recovery, fermentation, EtOH recovery and CHP were excluded from the preliminary techno-economic evaluation.

**Table 1:** Market prices of the feedstock, utilities and products.

	Cost (£)	Units
Seed-based *Mx2779* hybrid^a^	26.00	£/tonne
Novozymes Cellulase blend (Cellic CTec2)^b^	0.07	£/L EtOH
Novozymes *endo*-xylanase (NS22083)^c^	8.000.00	£/tonne
[TEA][HSO_4_] solvent (optimistic case of 99.9 % solvent recovery)^d^	12.80	£/tonne
[C_2_mim][OAc] solvent (very optimistic)^e^	26.00	£/tonne
Anti-solvent (tri-potassium phosphate)^f^	160.00	£/tonne
Electricity	0.10	kWh
Demineralised water	1.10	£/m3
Milling and grinding biomass (<10 % moisture content and 2 mm screen)	111.00	kWh/tonne

XOS^g^	3.000.00	£/tonne
Lignin (heating value)	130.00	£/tonne
Acetic Acid	480.00	£/tonne
EtOH	0.72	£/L

a Price includes an estimate for crop propagation, establishment, harvest by chipping and transportation.

b A 2 to 3 % (w/w) Cellic CTec2 loading and 20 % (w/w) solids loading was assumed.

c Recommended *endo*-xylanase dosage (0.25 % w/w total solids) was obtained from Novozymes.

d For the [TEA][HSO_4_] base case, 10 kg biomass and 1 kg IL loading was assigned.

e For the [C_2_mim][OAc] base case, 30 % (w/w) biomass and 70 % (w/w) IL loading was assigned.

f A 60 % (w/w) anti-solvent loading and 80 % anti-solvent recovery was assumed.

g A 71 % XOS recovery and purity greater than 91 % was assumed.

## 3 Results and discussion

### 3.1 Effects of pretreatment on biomass and component recovery

The impact of the sole and sequential SE and IL pretreatment on *Miscanthus Mx2779* biomass and component recovery are summarised in Table 2. The amount of recovered solids after pretreatment with [TEA][HSO_4_] (~56 %) and [C_2_mim][OAc] (~50 %) was in line with previous lab- and bench-scale studies (Brandt-Talbot et al., 2017, Li et al., 2010, Li et al., 2013). The mass loss of the starting material was attributed to the solubilisation of the cell wall components xylan, lignin, acetyl and other extractives during IL pretreatment (Table 2). Both SE and IL pretreatment itself and combinatorial SE + IL pretreatment of *Mx2779* resulted in high recovery of residual glucan (~85 to 99 %) relative to initial glucan of untreated and SE pretreated *Mx2779*, respectively, indicating that the applied pretreatment conditions preserved most of the glucan. [TEA][HSO_4_] pretreatment of *Mx2779* recovered ~85 % of the initial glucan (Table 2) and similar amounts of glucan remaining in the pulp are well-documented for [TEA][HSO_4_] pretreated *Miscanthus × giganteus* (*Mxg*) (Brandt-Talbot et al., 2017, Gschwend et al., 2016, Tu et al., 2020). Sole SE and [C_2_mim][OAc] pretreatment or combinatorial [SE] + [C_2_mim][OAc] pretreatment resulted in glucan recovery of ~86 to 91 % due to the dissolution of glucan into *gluco*-oligosaccharides (GOS), based on HPAEC analysis of the SE hydrolysate and [C_2_mim][OAc] wash solution. The dissolution of glucan (9 to 14 %) via SE, [C_2_mim][OAc] or [SE] + [C_2_mim][OAc] pretreatment (Table 2) is due to the removal of amorphous (less ordered/crystalline) cellulose and glucose from mixed-linkage glucan initially present in *Miscanthus* (Brandt-Talbot et al., 2017). These fractions were also most probably dissolved and removed by the [TEA][HSO_4_] pretreatment of *Mx2779* (Table 2).

**Table 2:** Effect of pretreatment on the mass balance of biomass components.

Conditions	Glucan	Xylan	Arabinan	Galactan	Lignin^b^	Acetyl	Others^c^	Recovered solids/pulp	Glucan recovery in solids/pulp	Xylan removal	Delignification	Deacetylation
	(w/w %)	(w/w %)	(w/w %)	(w/w %)	(w/w %)	(w/w %)	(w/w %)	(w/w %)	(%)	(%)	(%)	(%)
Untreated^a^	36.4 ± 1.0	19.5 ± 0.5	2.8 ± 0.2	1.1 ± 0.1	22.1 ± 1.0	3.9 ± 0.2	14.1 ± 0.4	100 ± 3.5	–	–	–	–
(As-received basis)												
Untreated^a^	42.3 ± 1.2	22.8 ± 0.6	3.3 ± 0.2	1.3 ± 0.1	25.8 ± 1.2	4.6 ± 0.3	–	100 ± 3.6	–	–	–	–
(Extractives-free basis)												
Steam Explosion [SE]^a^	40.4 ± 0.5	6.7 ± 0.2	0.4 ± 0.0	0.1 ± 0.0	26.7 ± 0.8	1.6 ± 0.3	14.4 ± 0.7	91.3 ± 4.2	87.6 ± 1.0	72.7 ± 0.9	4.1 ± 0.5	63.3 ± 7.2
[TEA][HSO_4_]	36.1 ± 0.0	10.1 ± 0.2	0.0 ± 0.0	0.0 ± 0.0	9.3 ± 0.0	0.0 ± 0.0	0.8 ± 0.2	56.4 ± 0.0	85.4 ± 0.1	55.8 ± 0.7	63.8 ± 0.1	100 ± 0.0
[SE] + [TEA][HSO_4_]	40.2 ± 0.2	3.6 ± 0.1	0.0 ± 0.0	0.0 ± 0.0	8.3 ± 0.3	0.0 ± 0.0	0.4 ± 0.0	52.5 ± 1.0	99.6 ± 0.5	46.8 ± 0.9	68.8 ± 1.1	100 ± 0.0
[C_2_mim][OAc]	31.4 ± 0.3	2.3 ± 0.2	0.9 ± 0.1	0.3 ± 0.0	5.8 ± 0.4	2.3 ± 0.0	7.0 ± 0.0	49.9 ± 1.1	86.2 ± 0.7	88.0 ± 0.8	73.7 ± 1.9	41.9 ± 1.1
[SE] + [C_2_mim][OAc]	36.8 ± 0.4	0.8 ± 0.0	0.0 ± 0.0	0.0 ± 0.0	8.8 ± 0.3	1.3 ± 0.0	15.7 ± 0.0	63.4 ± 0.8	91.0 ± 1.0	87.4 ± 0.5	67.2 ± 1.2	16.7 ± 1.2

$\mathrm{Solids}\;\;re\mathrm{cov}ery\;\;\left(\%\right)=\frac{\mathrm{Mass}\;\;of\;\;biomass\;\;regenerated\;\;\left(DM\right)}{\mathrm{Mass}\;\;of\;\;initial\;\;biomass\;\;\left(DM\right)}\times 100$.

$\mathrm{Component}\;\;re\mathrm{cov}ery\;\left(\%\right)=\frac{\mathrm{Component}\;\;in\;\;pretreated\;\;biomass\;\left(w/w\;\;\%\right)}{\mathrm{Component}\;\;in\;\;initial\;\;biomass\;\left(w/w\;\;\%\right)}\times 100$.

$\mathrm{Component}\;\;removal\;\;\left(\%\right)=100-Component\;\;re\mathrm{cov}ery\;\left(\%\right)$.

a Values were taken from our previous study ^15^.

b Lignin is total Klason lignin (acid-soluble and acid-insoluble lignin).

c Includes extractives, ash and other solids.

In contrast, both IL and combinatorial SE and IL pretreatments solubilised variable amounts of xylan (~47 to 88 %), lignin (~64 to 74 %) and acetyl (~17 to 100 %), thereby producing recovered solids with lower levels of residual xylan (~1 to 10 w/w %), lignin (~6 to 9 w/w %) and acetyl (up to 2 w/w %) (Table 2). Of the two ILs, surprisingly, [C_2_mim][OAc] dissolved more xylan and lignin than [TEA][HSO_4_], even though [TEA][HSO_4_] is well known as a selective lignin-extracting IL (Brandt-Talbot et al., 2017). Xylan removal as well as delignification observed for [C_2_mim][OAc] and [SE] + [C_2_mim][OAc] pretreatment ranged from 87 to 88 % and 67 to 74 %, respectively (Table 2). Depending on the monolignol composition and biomass source, the glass-transition temperature of lignin can vary between 100 and 160 °C (Arora et al., 2010). It appears that the higher pretreatment temperature for [C_2_mim][OAc] (160 °C) facilitated lignin extraction (~74 %) from *Mx2779* compared to the [TEA][HSO_4_] (120 °C) delignification (~64 %), which subsequently may have played an important role in the dissolution of xylan (Table 2). Further design of experiments is needed to clarify optimal [C_2_mim][OAc] or [TEA][HSO_4_] pretreatment process conditions for *Mx2779*.

Upon end-hydrolysis with 4 % H_2_SO_4_, HPAEC analysis confirmed that ~90 % (w/w) of the initial xylan from untreated *Mx2779* material was dissolved as xylo-oligosaccharides (XOS) in the [C_2_mim][OAc] wash solution. Other [C_2_mim][OAc] pretreatment reports recovered disparate XOS yields (~33 to 82 %) in the liquid phase, albeit using different IL parameters and lignocellulosic grasses such as switchgrass and corn stover (Li et al., 2010, Li et al., 2011, Li et al., 2013). Nonetheless, these IL derived XOS may signify opportunities for prebiotics production and could potentially enhance the competitiveness of the [C_2_mim][OAc] pretreatment process in a lignocellulosic biorefinery (Baral and Shah, 2016, Lopes et al., 2019).

In comparison, [TEA][HSO_4_] or [SE] + [TEA][HSO_4_] pretreatment completely removed acetyl groups and more lignin (64–69 %) than xylan (47–56 %), which is in agreement with [TEA][HSO_4_] primarily being a lignin-extracting solvent (Brandt-Talbot et al., 2017). It is interesting to note that the effect of combined SE and IL pretreatment did not drastically promote glucan recovery, xylan removal or delignification (Table 2), highlighting the efficacy of these two ILs regardless of the pre-processed biomass substrates, i.e., SE pretreated material. Even so, a previous [C_2_mim][OAc] pretreatment scale-up evaluation study on switchgrass indicated that process configurations such as a 5-fold higher biomass loading from 3 to 15 % (w/w) could result in less removal of xylan or recovery of lignin in the pulp stream (Li et al., 2013). The high lignin content (~27 w/w %) in the SE pretreated solids, when compared with untreated biomass (~22 w/w %), was attributed to the removal of a considerable amount of xylan (~73 %) and acetyl groups (~65 %) while retaining most of the lignin (Table 2).

### 3.2 Pretreatment impact on cellulose and lignin-related properties

The effects of ILs and combinatorial SE and IL pretreatment on lignocellulosic properties was further investigated by FTIR analysis. FTIR peak ratios between 897 cm^−1^ (β-(1,4) glycosidic bond in cellulose) and 1423 cm^−1^ (crystalline structure of cellulose), 1320 cm^−1^ (C–H rocking of glucose ring) and 3400 cm^−1^ (O–H stretching, H-bonds between molecules), and 1508 cm^−1^ (deformation of lignin CH_2_ and CH_3_) and 1600 cm^−1^ (stretching of C=C and C=O aromatic lignin) are described as lateral order index (LOI), also known as an empirical crystallinity index of cellulose, hydrogen bond intensity (HBI) and cross-linked lignin (CLL), respectively (Auxenfans et al., 2017).

Each sole pretreatment resulted in significantly lower LOI values compared with untreated *Mx2779* (Table 3). The substantially lower LOI values for SE, [TEA][HSO_4_] and [C_2_mim][OAc] are likely linked to shortened cellulose microfibrils or the allomorphic transformation of native parallel-packed arrays of cellulose microfibrils (cellulose I) to a mixture of antiparallel-packed fibrils of cellulose II and amorphous cellulose (Cheng et al., 2011, Tu et al., 2020). The little to no change in LOI values for combinatorial SE + ILs pretreatment may reflect evidence of remnant cellulose I or a paracrystalline regenerated cellulose II structure. These results may also indicate that the SE + IL pretreated *Mx2779* samples contain more organised and packed cellulose chains than the [TEA][HSO_4_] and [C_2_mim][OAc] pretreated samples. Compared to the low LOI value for SE pretreated *Mx2779*, a higher LOI value was reported for SE pretreated *Mxg* (Auxenfans et al., 2017). Glucan recovery was also reported higher for *Mxg* (~95 %) than *Mx2279* (~88 %) after similar SE conditions but with variable enzymatic glucose yields of ~ 40 and 70 % for *Mxg* and *Mx2779*, respectively (Bhatia et al., 2020). Overall, these results suggest that *Mxg* contains a more ordered and crystalline cellulose structure with lower accessibility to cellulase enzymes than *Mx2779* after SE pretreatment. These differences may originate from distinct biomass features between *Mx2779* and *Mxg* (Bhatia et al., 2020), thereby highlighting the importance of breeding or genetically engineered *Miscanthus* hybrids better suited for biorefining applications (da Costa et al., 2019).

**Table 3:** Effect of pretreatment on cellulose and lignin-related properties.

Conditions	LOI (A_1423_/A_897_)	HBI (A_3400_/A_1320_)	CLL (A_1508_/A_1600_)
Untreated	0.73 ± 0.01	3.29 ± 0.06	0.69 ± 0.04
[SE]	0.63 ± 0.03**	3.07 ± 0.06**	0.86 ± 0.03***
[TEA][HSO_4_]	0.58 ± 0.06**	4.08 ± 0.32***	0.78 ± 0.04**
[SE] + [TEA][HSO_4_]	0.72 ± 0.03	3.16 ± 0.07*	0.90 ± 0.02***
[C_2_mim][OAc]	0.67 ± 0.01***	4.18 ± 0.10***	0.93 ± 0.01***
[SE] + [C_2_mim][OAc]	0.70 ± 0.02	3.99 ± 0.12***	0.99 ± 0.01***

LOI, lateral order index; HBI, hydrogen bond intensity; CLL, cross-linked lignin. Data are expressed as means ± standard deviation (n ≥ 3). Student’s *t*-test (two-tail): *P ≤ 0.05; **P ≤ 0.01; ***P ≤ 0.001.

The HBI was significantly lower for SE and [SE] + [TEA][HSO_4_] pretreated solids compared with untreated *Mx2779* material (Table 3). These findings may indicate that the SE and [SE] + [TEA][HSO_4_] cellulose-rich pretreated pulp has a lower degree of intermolecular regularity or hydrogen bonds (Oh et al., 2005), which might be attributed to disruption and shortened DP of the cellulose (Tu et al., 2020). Pretreatment of *Mx2779* with either [TEA][HSO_4_], [C_2_mim][OAc] or [SE] + [C_2_mim][OAc] resulted in a higher HBI between neighbouring cellulose chains than untreated *Mx2779*, indicative of an increase in the inter-and intra-chain hydrogen bonding or a more organised cellulose structure. The FTIR peak intensity at 1740 cm^−1^ attributed to acetyl and uronic ester groups of the hemicellulose or from the ester linkage of the ferulic and *p*-coumaric acids of lignin and hemicellulose (Sun et al., 2005), suggested that ester linkages were not completely dissociated during SE, [C_2_mim][OAc] or [SE] + [C_2_mim][OAc] pretreatment. Indeed, SE pretreatment generated only low concentrations of *p*-coumaric and ferulic acid, as well as vanillin and syringaldehyde derived from the degradation of lignin (Bhatia et al., 2020). This is also consistent with the acetyl content in the recovered pretreated pulps (Table 2). The findings that acetyl-residues (~4 % w/w) were removed entirely during [TEA][HSO_4_] or [SE] + [TEA][HSO_4_] pretreatment (Table 2) tally with a previous study on [TEA][HSO_4_] pretreatment of *Mxg* which demonstrated that acetyl solutes of up to ~ 4 % (w/w) did not have a detrimental impact on [TEA][HSO_4_] reusability or effectiveness (Brandt-Talbot et al., 2017). Moreover, the authors also demonstrated that acetic acid could be separated from [TEA][HSO_4_] to provide an easily recoverable and valuable co-product stream.

The proportion of lignin with condensed and cross-linked structures, also known as cross-linked lignin ratio (CLL), was significantly increased after each pretreatment (Table 3), indicative of enrichment in lignin with condensed and cross-linked G-monomer lignin structures (Auxenfans et al., 2017, Brandt-Talbot et al., 2017). These modifications suggest residual lignin is still bound to polysaccharides via lignin-carbohydrate complexes, which matches with the 1740 cm^−1^ FTIR peak attribution as well as the residual lignin and xylan recovered in the pretreated pulp (Table 2). No increase in acid-soluble lignin content was observed for any of the pretreated solids, which suggests limited lignin fragmentation occurred after each pretreatment. Moreover, a significant negative correlation was found between the lignin parameter CLL and xylan content, whereby CLL values increased as xylan content decreased in the pretreated solids. A possible explanation for this may be that lignin and xylan are solubilised into the liquid phase during pretreatment and lignin is then repolymerised or condensed back onto the surface of the cellulose-enriched pretreated pulp (Auxenfans et al., 2017, Yoo et al., 2020). Such reactions, i.e., degree of condensation or repolymerisation of lignin, are not desirable for lignin valorisation processes and lignin-derived end products (Yoo et al., 2020). A detailed characterisation of lignin subunit composition and ether linkages, phenolic and carboxylic hydroxyl (OH) content as well as the molar mass of lignin (Gschwend et al., 2018), would shed further light on the suitability of the isolated lignin fractions for value-added applications.

### 3.3 Enzymatic hydrolysis of pretreated solids

**Fig. 1:**
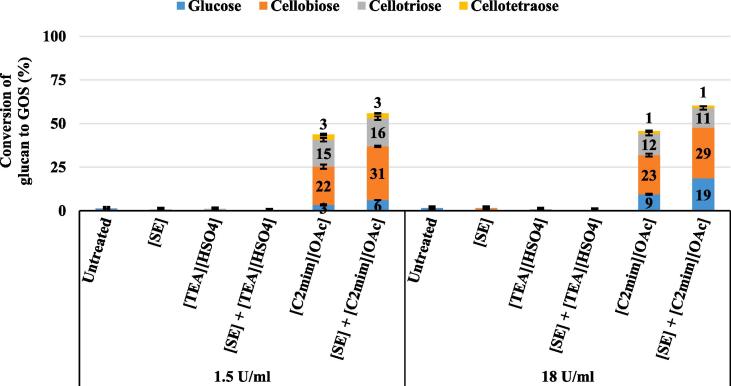
Conversion of glucan to glucose and GOS from untreated and pretreated *Miscanthus Mx2779* solids after 72 h of enzymatic hydrolysis with *endo*-1,4-β-D-glucanase at loadings of 1.5 U/mL and 18 U/mL.

To further investigate changes in cellulose structure and crystallinity, the cellulose-enriched pretreated pulp was hydrolysed using a commercial *endo*-1,4-β-D-glucanase that selectively digests amorphous regions of cellulose to release mainly GOS while preserving the more recalcitrant crystalline cellulose regions (Fig. 1). Most notably, *endo*-glucanase hydrolysis of the [C_2_mim][OAc] and [SE] + [C_2_mim][OAc] pretreated pulp enabled the production of ~ 40 to 50 % of available glucan into water-soluble GOS with different DP, i.e., cellobiose, cellotriose and cellotetraose (Fig. 1), indicative of an abundance in amorphous cellulose regions and cellulose II (Cheng et al., 2011). The cellobiose (~50 to 60 % of total GOS) and glucose (~3 to 6 %) was enzymatically released as glucan hydrolysis by-products (Fig. 1). Besides, [SE] + [C_2_mim][OAc] pretreated pulp exhibited ~20 % higher GOS yields than the [C_2_mim][OAc] pretreated pulp (Fig. 1), likely caused by factors such as the DP, molecular structure or smoother and accessible surface area of cellulose II (Bansal et al., 2012). Increasing the *endo*-glucanase loading (12-fold) did not improve GOS yields (Fig. 1), indicative that neither enzyme saturation nor inactivation was the reason for the incomplete glucan hydrolysis to GOS. Additionally, most *endo*-glucanase produce ~60 to 70 % soluble reducing sugars (mainly cellobiose) and ~30 to 40 % insoluble reducing sugars (longer than cellohexaose) (Wilson and Kostylev, 2012). Hence, the observed incomplete glucan hydrolysis to GOS with *endo*-glucanase (Fig. 1) is more plausibly related to the structural properties of the cellulose, which are changing during hydrolysis (Kluge et al., 2019). Nonetheless, the characteristics and full applications potential of the *endo*-glucanase obtained GOS require further investigation.

We observed no significant correlation between the *endo*-glucanase derived GOS yields and the cellulose-related parameters (LOI and HBI), the lignin parameters (CLL or content) or reduced xylan content of pretreated solids. These results would further suggest that the released GOS yields are associated with the level of transformation of native crystalline cellulose I to a mixture of cellulose II and amorphous cellulose. The negligible amounts of glucose and cellobiose released from SE, [TEA][HSO_4_] and [SE] + [TEA][HSO_4_] pretreated solids after *endo*-glucanase hydrolysis (Fig. 1) suggests an inaccessible cellulose surface and that these pretreatments may have retained the native cellulose fibre orientation and crystalline cellulose I lattice structure (Tu et al., 2020). These results further confirm that both ILs have a fundamentally different effect on the recovered cellulose properties, making the pulps potentially suitable for various biomaterial applications in the fields of packaging, food, cosmetics and medical products.

**Fig. 2:**
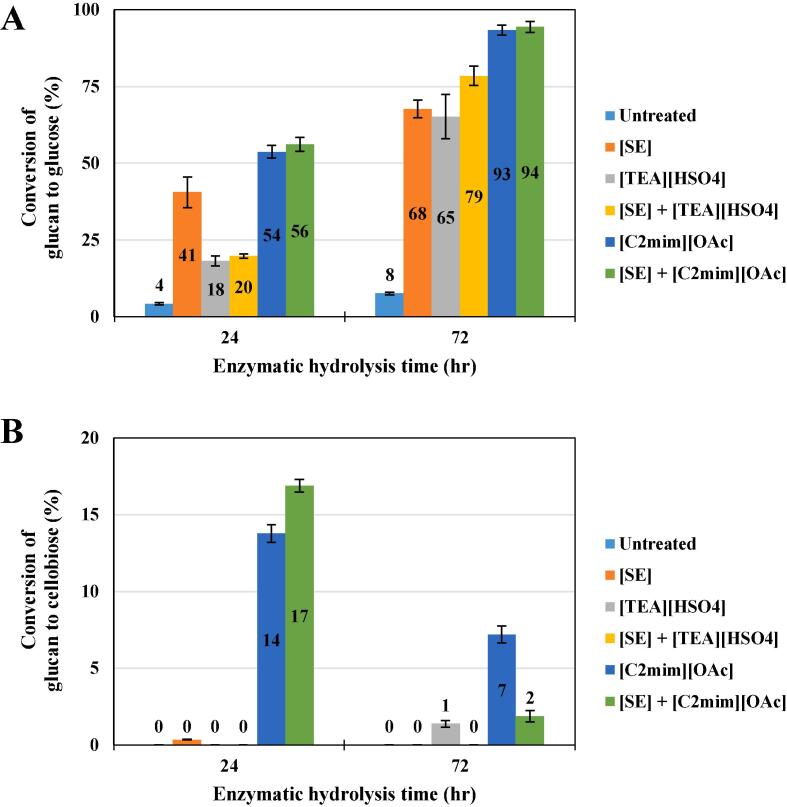
Conversion of glucan to (A) glucose and (B) cellobiose from untreated and pretreated *Miscanthus Mx2779* solids after 24 h and 72 h of enzymatic hydrolysis with the cellulase blend Cellic CTec2.

Enzymatic hydrolysis of the cellulose-rich pretreated pulp was also assessed using a high Cellic CTec2 dosage (30 % w/w g enzyme/g glucan) and a low biomass loading (1 % w/v) (Fig. 2). Although these hydrolysis factors do not provide an industrially relevant target, the primary goal of this assay was to determine how much cellulose could be converted into glucose for downstream fermentation. Fig. 2 shows the glucan digestibility profile into glucose and cellobiose after 24 and 72 h of enzymatic hydrolysis. The maximum glucose release from initial glucan of untreated *Mx2779* was 8 % after 72 h, reflecting low glucan digestibility of its recalcitrant biomass. While ~ 55 % of available glucan in [C_2_mim][OAc] and [SE] + [C_2_mim][OAc] pretreated *Mx2779* was hydrolysed into glucose after 24 h, ≤41 % of glucose was released for SE, [TEA][HSO_4_] and [SE] + [TEA][HSO_4_] pretreated solids within the same time interval (Fig. 2A). Interestingly, the glucose yields for [SE] + [TEA][HSO_4_] were lower than SE pretreated solids by a factor of ~2 after 24 h (Fig. 2A). The structural properties of their cellulose-enriched pulps could be the reason for the hydrolysis slow-down, as time-dependent enzyme deactivation or jamming effects slowing down hydrolysis were unlikely to have occurred due to the high Cellic CTec2 dosage (30 % w/w g enzyme/g glucan) and low pulp loading (1 % w/v). We also noted 14 to 17 % of glucan conversion to cellobiose (Fig. 2B) for [C_2_mim][OAc] and [SE] + [C_2_mim][OAc] pretreated solids, respectively, increasing the total glucan digestibility to ~70 % after only 24 h (Fig. 2).

Trace amounts (≤5 %) of [C_2_mim][OAc] are anticipated to co-precipitate with recovered cellulose due to IL intercalation between cellulose fibrils even after excessive washing of pulp (Li et al., 2013, Samayam et al., 2011). Moreover, several reports demonstrated that residual [C_2_mim][OAc] (≥5 %) is inhibitory and toxic to both cellulases and downstream fermentation (Mehmood et al., 2015, Ouellet et al., 2011, Park et al., 2012). Since we employed a high Cellic CTec2 dosage and low pulp loading (1 % w/v), it is improbable that the glucan digestibility was inhibited by enzyme related reasons and high residual [C_2_mim][OAc] present in the pretreated solids. Nonetheless, future research will focus on investigating lower [C_2_mim][OAc] usage and cellulase blend dosage levels and their compatibility with steps of the bioethanol production process, particularly with the enzymatic hydrolysis and fermentation phase. Despite that, discovering and bioengineering novel IL-tolerant cellulases also represents a strategy to improve the activity of cellulolytic enzymes and costs associated with excessive washing of the regenerated cellulose-enriched pulp for large-scale [C_2_mim][OAc] pretreatment applications (Datta et al., 2010).

With the help of SE, the conversion of glucan to glucose of the [SE] + [TEA][HSO_4_] pretreated pulp (79 %) was boosted by ~ 20 % after 72 h in comparison to [TEA][HSO_4_] pretreatment (65 %) (Fig. 2A). A recent [TEA][HSO_4_] pretreatment study on *Mxg* achieved optimum glucose yields of 72 %, though after a longer enzymatic hydrolysis time (168 h) and a lower Cellic CTec2 dosage (~20 % w/w g enzyme/g glucan) (Tu et al., 2020). Since cellulose crystallinity was shown to remain high for [TEA][HSO_4_] and [SE] + [TEA][HSO_4_] pretreated pulp (Fig. 1), the main cause for enhanced glucose yields could be ascribed mainly to the removal of xylan thus exposing the cellulose fibrils surface area to hydrolysing enzymes. No correlations were found in this study between the content of acetyl and lignin and the glucose yields, suggesting that de-lignification and -acetylation *per se* did not necessarily render the pretreated pulp more susceptible to enzymatic attack. Unlike [TEA][HSO_4_] and [SE] + [TEA][HSO_4_], glucan conversion to glucose for [C_2_mim][OAc] and [SE] + [C_2_mim][OAc] pretreated solids was ~95 % after 72 h (Fig. 2A), confirmative of a highly digestible cellulose pulp. Therefore, the drive of transformation from cellulose I to cellulose II represents a factor for improved enzymatic hydrolysis of the [C_2_mim][OAc] pretreated solids. Future experiments involving a higher *Mx2779* biomass loading (≥15 w/w %) and shorter pretreatment time (≤3 h) require an assessment to potentially overcome the usage drawbacks of [C_2_mim][OAc] due to its current high bulk cost (≥38 £/kg), compared to those for [TEA][HSO_4_] (~0.6 £/kg) (Baaqel et al., 2020, George et al., 2015, Liang et al., 2019). Nonetheless, the ~20 to 40 % higher glucose yields for [C_2_mim][OAc] compared to [TEA][HSO_4_] is consistent with previous reports (Li et al., 2010, Li et al., 2013, Sant’Ana da Silva et al., 2011). The significant correlation observed between xylan content, CLL and glucose hydrolysis yields imply that the high glucan to glucose conversion for [C_2_mim][OAc] pretreated samples was not only coupled to their low cellulose crystallinity (Fig. 1) but may have also synergistically been affected by the removal of xylan and enrichment in CLL (Auxenfans et al., 2017, Chen et al., 2020).

### 3.4 Preliminary techno-economic evaluation

Several competing pretreatment routes are available to produce oligosaccharides from various lignocellulosic materials. However, very few studies preliminarily assess the potential bio-based products revenue against the operational costs of their respective pretreatment process to determine any return on investments before estimating capital expenditures for commercial reality. Table 4 summarises the total product revenue and operating costs calculated for the SE and IL pretreatment processes of *Mx2779*. Among the list of value-added bio-products, xylan derived XOS was found to be a major contributor to product revenue accounting for ~68 % of total income, followed by EtOH (glucan), CHP (lignin) and acetic acid (acetyl). The gross margin can be high especially for XOS recovery when comparing the market prices of the high-value co-products (XOS: ~3000 £/tonne, cellulose pulp: ~192 £/tonne, lignin: ~130 £/tonne, acetic acid: ~480 £/tonne), and could contribute to the economic viability of an integrated biorefining process (Lopes et al., 2019). On the other hand, utility cost was a key contributor (~25 to 70 %) to total operating costs for SE and IL pretreatment processes, followed by biomass feedstock and other input material costs such as anti-solvent, IL and enzymes. The estimated *Mx2779* feedstock cost (~26 £/tonne) was ~3-fold lower than *Miscanthus Mxg* (~75 £/tonne) (Hastings et al., 2017). This was mainly due to a ~50 % reduction in crop establishment cost estimated for direct seed drilling of *Mx2779* compared to rhizome-propagated *Mxg*, as well as ~30 % lower cost associated with harvesting by direct biomass chipping instead of baling (Hastings et al., 2017). Therefore, the use of seed-based *Miscanthus* hybrids could be vital to economic and technical sustainability and add increased value to the overall production chains.

**Table 4:** Economic estimates to produce oligosaccharides and value-added bio-based products from 1 tonne of *Miscanthus* (*Mx2779*) under SE and IL pretreatment processes.

	Pretreatment process				
	SE	[TEA][HSO_4_]	SE + [TEA][HSO_4_]	[C_2_mim][OAc]	SE + [C_2_mim][OAc]
Revenue					
EtOH	100	106	120	134	124
Acetic Acid	6	17	16	10	11
Lignin (CHP)	88	83	79	78	75
XOS	286		286	336	286
Total revenue (£)	480	206	501	558	496

Variable operating costs					
Feedstock	26	26	26	26	26
Feedstock (milling + grinding)		11		11	
Enzymes (Cellic CTec2)	10	10	12	13	12
Enzymes (*endo*-xylanase)	2		2		2
IL		13	13	26	26
Anti-solvent				77	77
Utilities (water + electricity)	78	44	122	51	129
Total operating costs (£)	116	104	175	204	272

Net (£)	364	101	326	354	224
Gross margin (%)	76	49	65	63	45

EtOH, Ethanol; CHP, combined heat and power; XOS, xylo-oligosaccharides; IL, ionic liquid.

In terms of process consumables, the anti-solvent and IL were amongst the major contributors (~38 %) to the operating costs of [C_2_mim][OAc] pretreatment (Table 4), even with a very favourable assumed price for [C_2_mim][OAc] (~26 £/tonne) and anti-solvent (~160 £/tonne) at the biorefinery gate (Baral and Shah, 2016). Most reported literature for [C_2_mim][OAc] pretreatment studies generally focuses on the liberation of fermentable monomeric sugars (glucose and xylose) from biomass. However, the present studies approach could reduce overall process costs by directly providing higher-value XOS or GOS. Separation of the oligosaccharides from the dilute aqueous mixture of [C_2_mim][OAc] and near-complete recovery of [C_2_mim][OAc] still pose technical, economic and sustainability challenges and must be further addressed to make this IL pretreatment process viable.

Biomass conventionally requires milling for particle size reduction before IL pretreatment to ensure even IL to particle contact and uniform biomass fractionation. Hence, combining SE + IL pretreatment could help circumvent operating costs associated with mechanical biomass comminution, can more effectively deconstruct biomass, and may also further reduce operating costs for downstream purification of dissolved sugars from the IL solution and recovery and reusability of ILs. In this regard, the complementarity of SE with [TEA][HSO_4_] pretreatment suggested a ~ 33 % profit enhancement relative to [TEA][HSO_4_] alone, mainly due to recovery of XOS derived via the SE pretreatment process step (Table 4). It should be noted that a previous preliminary techno-economic evaluation of [TEA][HSO_4_] pretreatment for the fractionation of *Mxg* indicated furfural (800 £/tonne) as a major contributor to product revenue accounting for ~43 % of total revenue (Brandt-Talbot et al., 2017). This is because [TEA][HSO_4_] dissolves the hemicellulose fraction mainly into monomeric form, and the pentoses (xylose and arabinose) are partially converted into furfural. Whilst this was not part of the present study, the co-production of furfural from the combined SE + [TEA][HSO_4_] pretreatment process merits further investigation to assess optimal process integration and maximise the cost-efficient biomass utilisation of *Mx2779*.

## 4 Conclusions

This study demonstrates that SE and IL pretreatment, alone or in combination, result in different outputs, suited for specific downstream bioprocesses and applications. [C_2_mim][OAc] might offer advantages over [TEA][HSO_4_] via potential XOS recovery and leaving behind a highly enzymatically digestible cellulose pulp for biofuel production or potential GOS-based product applications. The complementarity of SE with [TEA][HSO_4_] pretreatment and co-production of xylan-based XOS may open a promising path to further improving overall techno-economics of [TEA][HSO_4_] pretreatment. Bench- and pilot-scale investigations, along with detailed techno-economic analysis, are required to ensure the viability of such pretreatment process strategies for an integrated biorefinery.

## CRediT authorship contribution statement

**Rakesh Bhatia:** Validation, Investigation, Writing - original draft, Writing - review & editing, Visualization, Project administration. **Jai B. Lad:** Validation, Writing - review & editing, Visualization. **Maurice Bosch:** Validation, Writing - review & editing, Supervision. **David N. Bryant:** Validation, Writing - review & editing, Supervision. **David Leak:** Conceptualization, Validation, Writing - review & editing, Funding acquisition. **Jason P. Hallett:** Validation, Writing - review & editing. **Telma T. Franco:** Conceptualization, Validation, Writing - review & editing, Funding acquisition. **Joe A. Gallagher:** Validation, Writing - review & editing, Supervision, Funding acquisition.

## Declaration of Competing Interest

The authors declare that they have no known competing financial interests or personal relationships that could have appeared to influence the work reported in this paper.
