# Supplementary material for: Production of oligosaccharides and biofuels from Miscanthus using combinatorial steam explosion and ionic liquid pretreatment
Source: Bioresour Technol. 2021 Mar;323:124625. doi: 10.1016/j.biortech.2020.124625 (PMC7873588; doi:10.1016/j.biortech.2020.124625)
Supplement: Supplementary data 1 [file mmc1.pptx]

## Slide 1
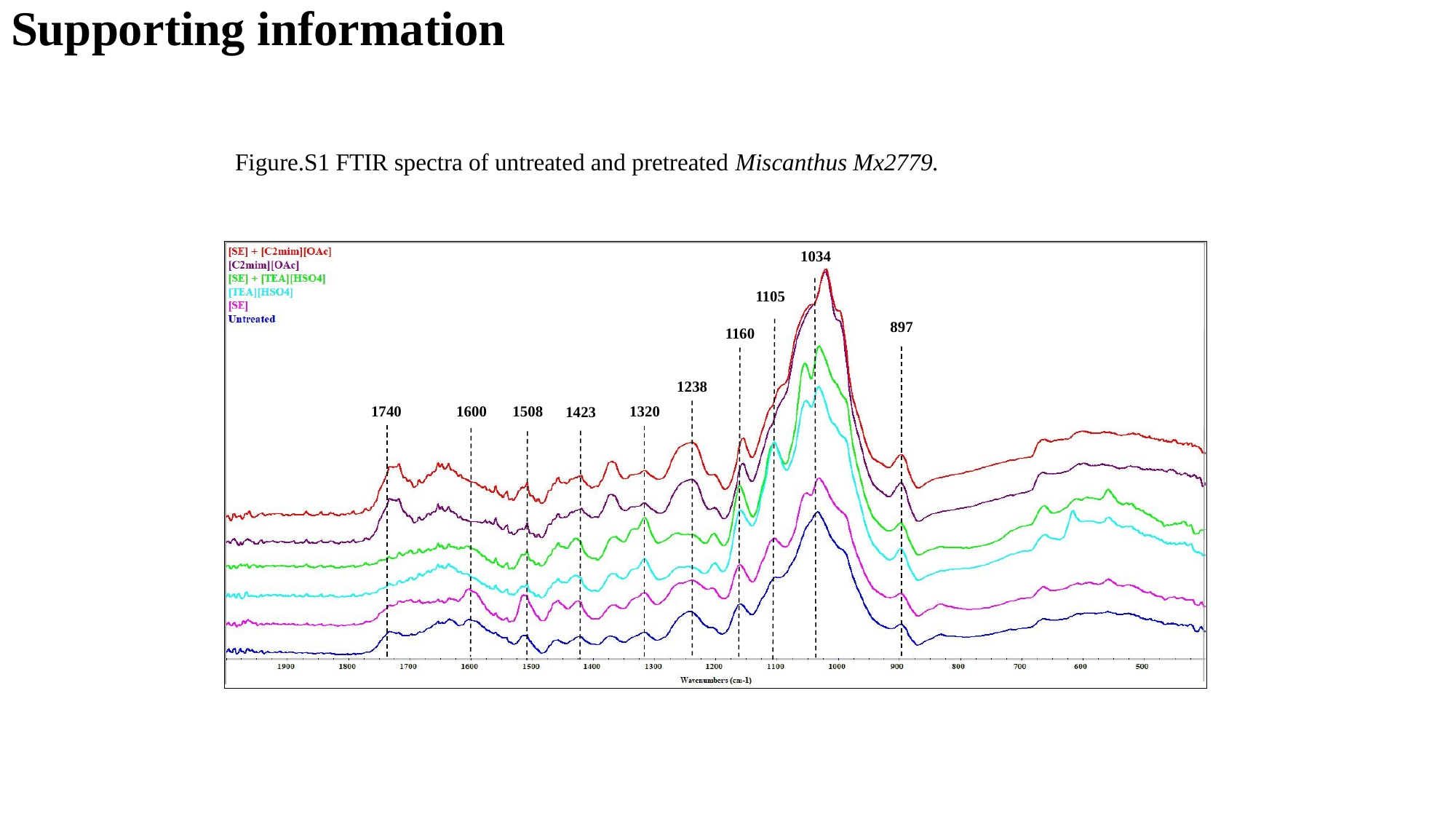

# Supporting information
Figure.S1 FTIR spectra of untreated and pretreated Miscanthus Mx2779.
1034
1105
897
1160
1238
1740
1600
1508
1320
1423

## Slide 2
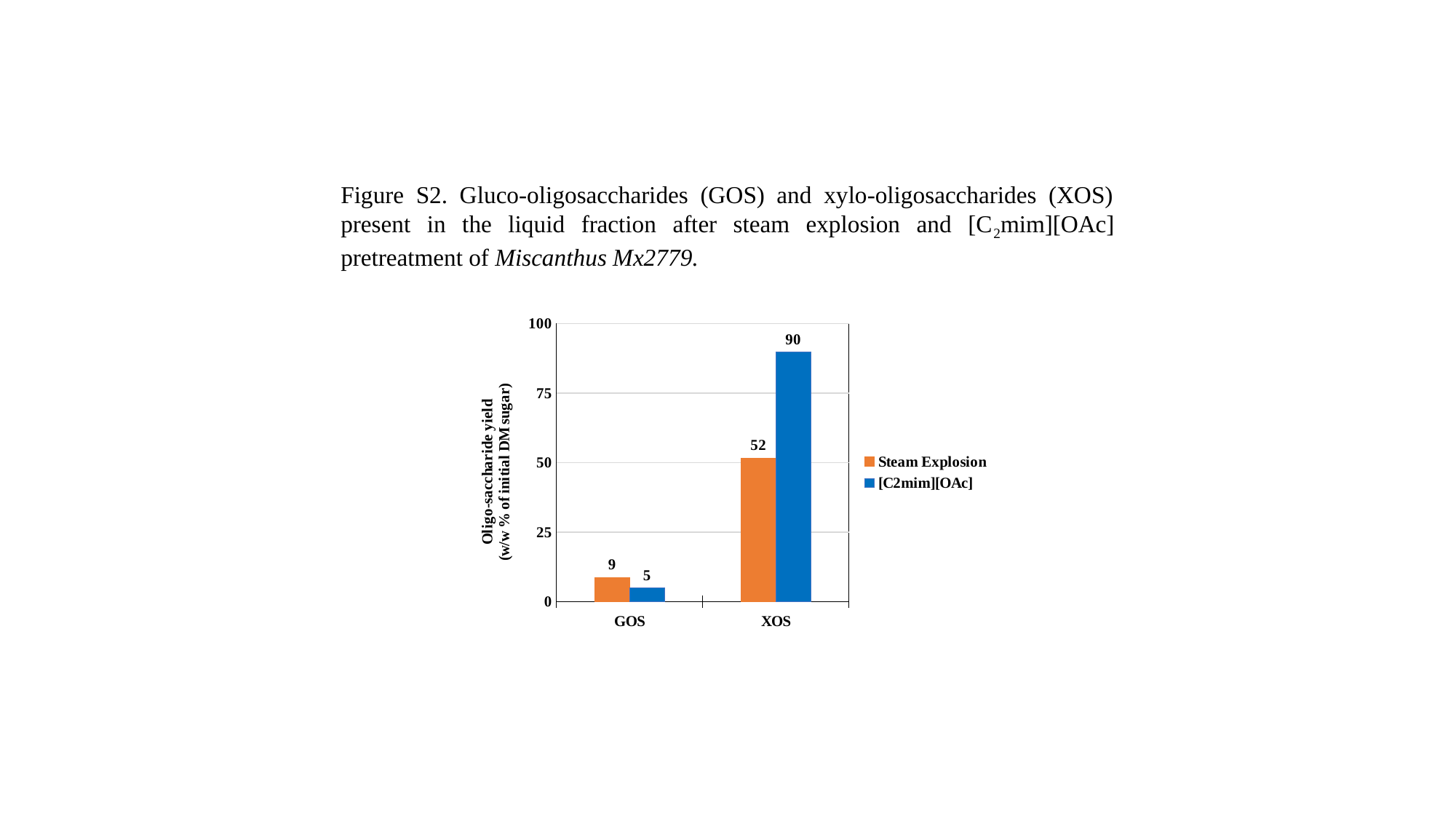

Figure S2. Gluco-oligosaccharides (GOS) and xylo-oligosaccharides (XOS) present in the liquid fraction after steam explosion and [C2mim][OAc] pretreatment of Miscanthus Mx2779.
### Chart
| Category | Steam Explosion | [C2mim][OAc] |
|---|---|---|
| GOS | 8.749709848789262 | 4.971423517159896 |
| XOS | 51.620112103122395 | 89.72826756763426 |

## Slide 3
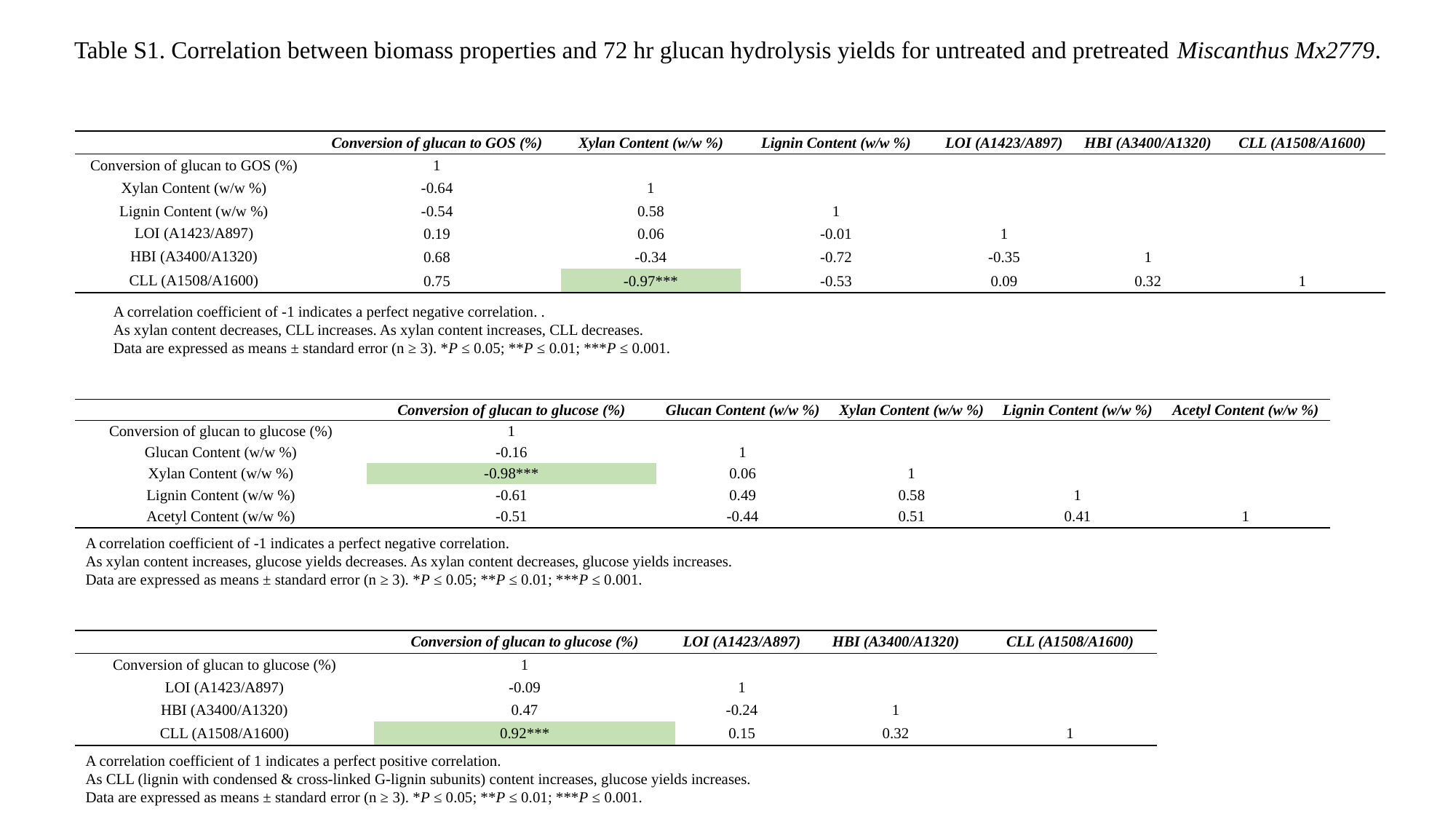

Table S1. Correlation between biomass properties and 72 hr glucan hydrolysis yields for untreated and pretreated Miscanthus Mx2779.
| | Conversion of glucan to GOS (%) | Xylan Content (w/w %) | Lignin Content (w/w %) | LOI (A1423/A897) | HBI (A3400/A1320) | CLL (A1508/A1600) |
| --- | --- | --- | --- | --- | --- | --- |
| Conversion of glucan to GOS (%) | 1 | | | | | |
| Xylan Content (w/w %) | -0.64 | 1 | | | | |
| Lignin Content (w/w %) | -0.54 | 0.58 | 1 | | | |
| LOI (A1423/A897) | 0.19 | 0.06 | -0.01 | 1 | | |
| HBI (A3400/A1320) | 0.68 | -0.34 | -0.72 | -0.35 | 1 | |
| CLL (A1508/A1600) | 0.75 | -0.97\*\*\* | -0.53 | 0.09 | 0.32 | 1 |
A correlation coefficient of -1 indicates a perfect negative correlation. .
As xylan content decreases, CLL increases. As xylan content increases, CLL decreases.
Data are expressed as means ± standard error (n ≥ 3). *P ≤ 0.05; **P ≤ 0.01; ***P ≤ 0.001.
| | Conversion of glucan to glucose (%) | Glucan Content (w/w %) | Xylan Content (w/w %) | Lignin Content (w/w %) | Acetyl Content (w/w %) |
| --- | --- | --- | --- | --- | --- |
| Conversion of glucan to glucose (%) | 1 | | | | |
| Glucan Content (w/w %) | -0.16 | 1 | | | |
| Xylan Content (w/w %) | -0.98\*\*\* | 0.06 | 1 | | |
| Lignin Content (w/w %) | -0.61 | 0.49 | 0.58 | 1 | |
| Acetyl Content (w/w %) | -0.51 | -0.44 | 0.51 | 0.41 | 1 |
A correlation coefficient of -1 indicates a perfect negative correlation.
As xylan content increases, glucose yields decreases. As xylan content decreases, glucose yields increases.
Data are expressed as means ± standard error (n ≥ 3). *P ≤ 0.05; **P ≤ 0.01; ***P ≤ 0.001.
| | Conversion of glucan to glucose (%) | LOI (A1423/A897) | HBI (A3400/A1320) | CLL (A1508/A1600) |
| --- | --- | --- | --- | --- |
| Conversion of glucan to glucose (%) | 1 | | | |
| LOI (A1423/A897) | -0.09 | 1 | | |
| HBI (A3400/A1320) | 0.47 | -0.24 | 1 | |
| CLL (A1508/A1600) | 0.92\*\*\* | 0.15 | 0.32 | 1 |
A correlation coefficient of 1 indicates a perfect positive correlation.
As CLL (lignin with condensed & cross-linked G-lignin subunits) content increases, glucose yields increases.
Data are expressed as means ± standard error (n ≥ 3). *P ≤ 0.05; **P ≤ 0.01; ***P ≤ 0.001.

## Slide 4
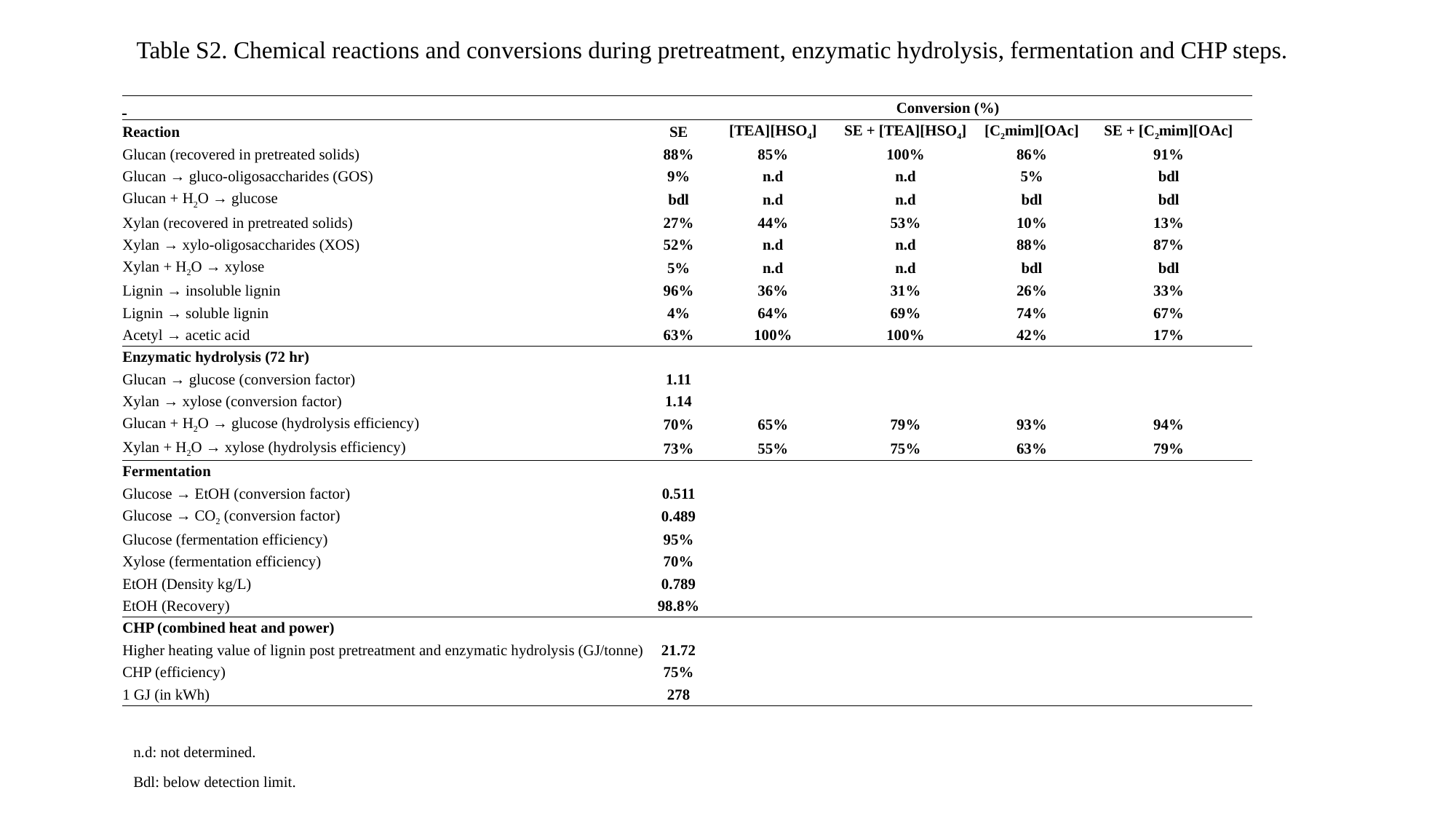

Table S2. Chemical reactions and conversions during pretreatment, enzymatic hydrolysis, fermentation and CHP steps.
| | Conversion (%) | | | | |
| --- | --- | --- | --- | --- | --- |
| Reaction | SE | [TEA][HSO4] | SE + [TEA][HSO4] | [C2mim][OAc] | SE + [C2mim][OAc] |
| Glucan (recovered in pretreated solids) | 88% | 85% | 100% | 86% | 91% |
| Glucan → gluco-oligosaccharides (GOS) | 9% | n.d | n.d | 5% | bdl |
| Glucan + H2O → glucose | bdl | n.d | n.d | bdl | bdl |
| Xylan (recovered in pretreated solids) | 27% | 44% | 53% | 10% | 13% |
| Xylan → xylo-oligosaccharides (XOS) | 52% | n.d | n.d | 88% | 87% |
| Xylan + H2O → xylose | 5% | n.d | n.d | bdl | bdl |
| Lignin → insoluble lignin | 96% | 36% | 31% | 26% | 33% |
| Lignin → soluble lignin | 4% | 64% | 69% | 74% | 67% |
| Acetyl → acetic acid | 63% | 100% | 100% | 42% | 17% |
| Enzymatic hydrolysis (72 hr) | | | | | |
| Glucan → glucose (conversion factor) | 1.11 | | | | |
| Xylan → xylose (conversion factor) | 1.14 | | | | |
| Glucan + H2O → glucose (hydrolysis efficiency) | 70% | 65% | 79% | 93% | 94% |
| Xylan + H2O → xylose (hydrolysis efficiency) | 73% | 55% | 75% | 63% | 79% |
| Fermentation | | | | | |
| Glucose → EtOH (conversion factor) | 0.511 | | | | |
| Glucose → CO2 (conversion factor) | 0.489 | | | | |
| Glucose (fermentation efficiency) | 95% | | | | |
| Xylose (fermentation efficiency) | 70% | | | | |
| EtOH (Density kg/L) | 0.789 | | | | |
| EtOH (Recovery) | 98.8% | | | | |
| CHP (combined heat and power) | | | | | |
| Higher heating value of lignin post pretreatment and enzymatic hydrolysis (GJ/tonne) | 21.72 | | | | |
| CHP (efficiency) | 75% | | | | |
| 1 GJ (in kWh) | 278 | | | | |
n.d: not determined.
Bdl: below detection limit.
